# New and old challenges for clinical psychology and psychotherapy toward 2030

**DOI:** 10.3389/fpsyg.2026.1789643

**Published:** 2026-05-29

**Authors:** Gianluca Castelnuovo

**Affiliations:** 1 Department of Psychology, Catholic University of Milan, Milano, Italy; 2 IRCCS Istituto Auxologico Italiano, Clinical Psychology Research Laboratory, Piancavallo, Italy

**Keywords:** artificial intelligence, clinical health psychology, clinical psychology, digital interventions, Internet interventions, personalized interventions, psychotherapy, stepped-care approach

## Introduction

The main goal of this grand challenge article is to look at current issues, opportunities, and trends about clinical psychology and psychotherapy fields, that include a wide range of topics related to counseling, rehabilitation psychology, neuropsychology, and all types of psychological interventions in both traditional clinical settings (like hospitals, clinics, services, laboratories, etc.) and new clinical ones (like remote outpatient clinics, telehealth, e-health, and mHealth-based settings). The synergic match of medicine and psychology in the interdisciplinary treatment of primary organic and mental diseases leads to a substantial progression of the biopsychosocial approach in contemporary clinical and research initiatives within the health care sector. Medicine alone could be considered “a soul without psychology” (TIME magazine—December 24, 1956), but fortunately now there is no medical area without a corresponding field in Clinical Psychology: psycho-cardiology, psycho-oncology, psycho-geriatrics, psycho-pneumology, psycho-endocrinology, psycho-neurology and neuropsychology, psychology in pain management, in surgery, etc., are only some applications of the significant impact of psychology into clinical settings. The Lancet's warning that “No health without mental health” (Prince et al., 2007) is still valid, as are the words “No medicine without psychology” (Castelnuovo, 2010b).

## The future of Clinical Psychological Investigation: where are we going? A tentative update

My previous “Grand Challenge” article on the clinical (and health) psychology specialist area of the Frontiers in Psychology journal (Castelnuovo, 2017) indicates that several of the suggested 10 major themes remain intriguing and hold potential as catalysts for study within our discipline.

First I will revisit the old challenges I identified in 2017 and then I will discuss the new challenges that have arisen since then, trying to find similarities but also novelties in the development of topics focused on clinical psychology fields.

1. *Integration between psychological treatments-psychotherapy and pharmacology* In 2017, I proposed that future research should move ahead from the traditional dichotomy between pharmacological and psychological treatments to an integrative framework, given the substantial evidence supporting the efficacy of combination therapy over monotherapy in the treatment of depression (Cox et al., 2014; Cuijpers et al., 2014; Guidi et al., 2011, 2016; Weitz et al., 2017, 2015). It is very relevant that further research will focus on identifying the optimal combination or sequential application of psychotherapy and pharmacotherapy for each specific psychopathology and individual patient (Guidi et al., 2016), while also taking into account the patient's preferences (Angermeyer et al., 2017). In recent years, numerous additional evidences have emerged about classic psychotropic medicines. Leichsenring et al. (2022) conducted a comprehensive umbrella review that reported evidence from 102 meta-analyses, incorporating approximately 3,800 randomized controlled trials and over 650,000 patients, to assess the efficacy of psychotherapies and pharmacotherapies for the most important adult mental disorders. When we only considered the best studies from a methodological point of view, both psychotherapies and pharmacotherapies had statistically significant effect sizes, but they were mainly minor. Direct comparisons underlined that there were relatively small differences between the two types of treatment, and combining them only gave a few extra benefits. This article well-underlined the “ceiling effect” in contemporary treatment research, noting that in spite of many years of disorder-specific research and development about interventions, minimal improvements have been achieved in real efficacy. Methodological limitations—such as publication bias, inadequate control conditions, brief treatment duration, and insufficient follow-up— further limit the interpretability and therapeutic significance of current research. Even if the statistically significant effects found in the article are below the threshold and some differences are not so relevant for a current clinical impact, for clinical psychology and psychotherapy it is necessary a paradigm change toward more inventive, individualized, and mechanism-oriented methodologies, taking into account the need of enhancing therapy specificity, tackling heterogeneity in treatment response, merging staging and personalizing tactics, and broadening research on long-term and preventive therapies. In order to improve the efficacy of psychotherapy, methodological precision and transdiagnostic perspectives, are requested in order to address all the complexities of real-world clinical practice.
  A recent Cochrane systematic review (Schipper et al., 2024) sheds light about the growing phenomenon of new psychedelic drugs, considering advantages and disadvantages of psychedelic-assisted therapy (PAT) for many mental disorders. Six randomized controlled studies with 149 people investigated conventional psychedelics (psilocybin and LSD) and MDMA administered inside established psychotherapy settings. Low-certainty data reported that traditional psychedelics may reduce anxiety and depressive symptoms at least in a short-term perspective, however evidence for MDMA remains highly uncertain. There were no major negative effects that happened throughout treatment, however mild to moderate short-term side effects were widespread. Even if the review has significant methodological limitations (limited sample sizes, inadequate blinding procedures, and expectation effects), these results open to new growing challenges in clinical psychology and psychotherapy: the integration of innovative new psychedelic drugs-based interventions into current clinical practice and the possible new combinations of psychotherapy, as an active mechanism of change, with new potential drugs in order to improve change effects. Other problems include making sure that therapists are well-trained and follow the rules, dealing with patients who are vulnerable when they are in altered states of consciousness, and finding a way about legal and regulatory restrictions because most psychedelics are still illegal. This review noted that psychedelic-assisted therapy may serve as a transformative intervention in palliative mental health care.
2. *Integration between psychological treatments-psychotherapy and neuroscience* Promoting research on neuroimaging and neuroscience could enhance our comprehension of psychological and psychopathological phenomena (Allen et al., 2017; Aybek and Vuilleumier, 2017; McArthur, 2017; Warren et al., 2017), as documented in Castelnuovo (2017). Recent developments have emerged, including a substantial multicenter diagnostic study (Lett et al., 2025) that introduced an innovative framework for psychopathology based on shared neurobiological mechanisms rather than conventional DSM/ICD-based diagnostic categories.
  Considering multimodal neuroimaging and symptomatology from both population based and clinical cohorts, the authors defined six transdiagnostic aspects of psychopathology that transcend traditional diagnostic categories and report strong correlations with specific brain-based signatures. The results underscored essential deficiencies in nosographic systems that lack biological insight, such as diagnostic variability, elevated comorbidity, and poor connections between diagnoses and their underlying mechanisms. This article highlights a significant future problem for clinical psychology and psychotherapy: present evaluation, case conceptualization, and treatment planning are predominantly diagnosis-driven, despite increasing evidence that symptoms aggregate along neurobiologically relevant dimensions. To apply brain-derived dimensions in clinical practice, we need to move away from disorder-specific procedures and toward dimensional, mechanism-focused models of care. Other problems include figuring out how to use complex neurobiological data in clinically useful assessment instruments, avoiding neuro-reductionism while keeping psychosocial formulation, and reaching an agreement on which biomarkers are legitimate and useful. The need of multimodal integration rather than dependence on singular biological markers is to take into account. In general, the study says that the future of clinical psychology and psychotherapy will depend on how well they can combine new discoveries in neuroscience with transdiagnostic, biopsychosocial, and person-centered models of care.
3. *Development of new areas of connection between clinical psychology and medicine not yet explored* As emphasized in 2017, next to traditional areas of collaboration between medicine and psychology such as psychocardiology (Ginsberg et al., 2015) or psychooncology (Castelli et al., 2015) or pain management (Castelnuovo et al., 2016a,b), areas of collaborations such as psychogeriatry, psycho-pneumology, psycho-endocrinology, or topics such as health behavior and prevention, psychosocial aspects of medical illness or impact of organic conditions on functioning have to be more deepened (Castelnuovo, 2017). In recent years, additional data has emerged across various medical domains, including promising new fields like as nephrology (Schmill et al., 2025).
4. *Integration of bio-physiological data with psychological ones*. As stated in the previous point 3, a psychosomatic approach examining the direct psychobiological effects of cognitions, emotions, behaviors, and psycho-social and relational factors on the pathophysiology of medical diseases remains an evolving area of research (Fava et al., 2014; Guidi et al., 2013). Furthermore, recent years have witnessed the emergence of mind-body and epigenetic investigations, providing additional data to the scientific and clinical domains (Jinich-Diamant et al., 2025; Kremer et al., 2025).
5. *Focus on positive psychology* The positive psychology paradigm (Huber, 2016; Schrank et al., 2014) may be especially effective in pre-clinical domains such as prevention and wellness (Zinzen et al., 2025), as well as in non-clinical groups (Laure et al., 2025).
6. *Integration of clinical psychological protocols with new technologies, monitoring strategies, mhealth, and virtual reality* In 2017, I stated that the use of mHealth platforms and new technologies could help clinicians in some critical situations such as the continuity of health assistance after a traditional period of inpatient care providing promising opportunities of monitoring and motivating patients, above all in the follow-up phase of treatment (Castelnuovo et al., 2016c) or with rural populations with limited access to health care services. mHealth must validate its efficacy, necessitating further research, especially in the realm of cost-effectiveness, where emerging technologies may play a distinct role in a stepped-care model (Castelnuovo, 2017; Castelnuovo et al., 2015b, 2016d; Castelnuovo and Simpson, 2011). The proliferation of validated and recognized digital therapies in recent years could be pivotal (Bertoli et al., 2025; Carrera et al., 2023; Castelnuovo et al., 2023), particularly in light of the impact of virtual reality-assisted interventions (Antichi et al., 2025; Oasi et al., 2025; Riva et al., 2023; Rossi et al., 2025). There will be a separate paragraph about the contribution of Artificial Intelligence has helped.
7. *Adapting clinical psychological protocols to special populations and contexts (chronic care management, elderly or active aging, immigrants, etc.)* Clinical psychology and psychotherapy have evolved and modified traditional protocols to address emerging populations and contexts, including chronic patients (Castelnuovo et al., 2015a,b), elderly individuals (Molinari et al., 2014), and immigrants, minorities, and refugees with customized care and treatment strategies (Dewitz et al., 2025; Mahajan et al., 2025).
8. *Study of mediators and moderators to change in clinical psychology and psychotherapy* Given that empirically supported treatments (ESTs) have already demonstrated their clinical efficacy within the evidence-based medicine (EBM) framework (Castelnuovo, 2010a), further investigation must concentrate on the examination of mediators and moderators, both within and beyond a Common Factors perspective, to identify or validate common or specific key elements for change and symptom reduction in clinical psychology and psychotherapy approaches (Holmbeck, 1997; Labus, 2007; Perz et al., 2011). A potential risk of conceptual defragmentation is outlined in the designated section.
9. *Empowering of assessment techniques in clinical health psychology* In 2017 (Castelnuovo, 2017), I stated that new questionnaires, scales, and semistructured interviews are necessary and must be implemented and validated to provide healthcare professionals with reliable and valid psychometric tools that have clinical significance (Belar et al., 2001; Joseph and Wood, 2010; Mihura et al., 2017; Riva et al., 2009). More study is needed in this area, taking into account the clinimetrics paradigm (Fava and Belaise, 2005; Horn et al., 2012; Mahendran, 2012; Streiner, 2003; Tomba and Bech, 2012). Machine learning, big data analysis, and AI-assisted algorithms can now help doctors with assessments and diagnoses. This includes deep learning, natural language processing, and digital phenotyping (Calderone et al., 2025).
10. *Delivering of guidelines and recommendations for more clinical impact-applications* In 2017 (Castelnuovo, 2017), I stated that to achieve a significant impact in the clinical community, research must fill the gap between theory and practice by providing “toolkits” for clinicians, such as guidelines or recommendations. The Italian Consensus Conference on Pain in Neurorehabilitation is an example that could be followed. It made specific suggestions in clinical health psychology (Aloisi et al., 2016; Castelnuovo et al., 2016a,b; Tamburin et al., 2016). Today, clinical protocols have been tailored to specific populations, and major international diagnostic manuals in mental health, such as DSM-5-TR and PDM-3, have enhanced their cultural sections to more accurately reflect not only Western society but also various cultures and subcultures.

## The role of Digital and AI-assisted interventions in clinical psychology and psychotherapy

Digital mental health interventions (DHIs) are becoming a relevant issue of current mental health approach, taking into account the widespread of mental illnesses. DHIs include different techniques, including mobile health applications, wearable technology, virtual and augmented reality, conversational agents, and artificial intelligence-driven systems. Many studies, overall about guided internet-based cognitive behavioral therapy, note that online therapies can have the same clinical effects as in-person treatments, but they are cheaper and easier to scale. But it is important to take into account that the quality of digital mental health tools varies a lot due to the lack of rigorous tests. Along with effectiveness, there are still key problematic doubts about data privacy, algorithmic transparency, ethical accountability, and fair access. This changing situation shows how important it is to have evidence-based frameworks that can help with research, implementation, and regulation of digital technologies in mental health treatment (Lochner et al., 2025).

More and more individuals are adopting large language models (LLMs) like ChatGPT for mental health reasons because traditional psychological care is hard to get because it's too expensive, there aren't enough physicians, people are ashamed to go, and they have to wait a long time. Although LLMs have demonstrated the capacity to provide contextually relevant and human-like therapeutic responses, empirical knowledge regarding users' actual experiences with these tools remains inadequate. Most prior research has focused on theoretical frameworks of AI in healthcare rather than empirical experiences of therapeutic implementation. To fill this gap, this article studies current user experiences with ChatGPT for mental health support by analyzing publicly accessible posts on Reddit, using a mixedmethods approach that matches qualitative theme coding with network analysis, and providing an empirically validated summary of the benefits, drawbacks, and risks associated with the use of ChatGPT in a therapeutic settings. In order to avoid these limitations, integration of big language models into future digital mental health ecosystems has to be gradual and cautious (Collins et al., 2025).

A recent relevant systematic review and multilevel meta-analysis (Meyer-Keirath et al., 2025) studied the comparability of video-based psychotherapy (VBT) to face-to-face (F2F) psychotherapy in reducing psychopathological symptoms in different mental disorders. Following PRISMA guidelines, randomized controlled trials directly comparing synchronous video-based therapy (VBT) and face-to-face (F2F) psychotherapy with a minimum treatment length of 500 min were incorporated. Eleven randomized controlled trials (RCTs) with more than 800 adult participants affected by PTSD, depression, anxiety disorders, obsessive-compulsive disorder, bulimia nervosa, and somatoform pain were included in the review. A three-level meta-analytic methodology was employed to tackle multiple outcomes per study and inter-study heterogeneity. There were no significant differences in posttreatment symptom severity between VBT and F2F psychotherapy across 36 symptom outcomes and no substantial moderating factors were identified. Most of the differences were because of changes between studies, not the type of treatment. The findings indicate that video-based psychotherapy can alleviate symptoms comparably to traditional face-toface therapy when conducted under rigorous methodological frameworks. These results support video-based interactions as a sustainable and effective approach for providing psychotherapy, but further research about long-term outcomes, underrepresented disorders, non-Western contexts, and non-inferiority trial methodologies is needed (Meyer-Keirath et al., 2025).

## From DSM-based categorial and nosographic approach to genetically oriented clinical psychology and psychotherapy interventions

Mental disorders have been traditionally considered and classified as distinct, episodic, and categorically independent conditions. Caspi et al. (2014) have underlined that, despite this mainstream perspective, psychopathological diseases frequently present chronic/recurrent patterns, significant substantial comorbidity and dimensional continuity across apparently different diagnostic categories. These authors examined the structure of psychopathology, through a longitudinal data methodology, analyzing symptom dimensionality, temporal persistence, co-occurrence, and sequential comorbidity from adolescence to midlife. Initial studies confirmed a hierarchical framework characterized by three higher-order dimensions: Internalizing, Externalizing, and Thought Disorder. However, the data were more clearly explained by a single and all-comprehensive component of general psychopathology. The p factor, like the g factor in IQ studies, can reveal how different psychopathological states can generate the same profiles: higher p factor scores were associated with more functional impairment and more adverse developmental histories, also with early indicators of compromised brain functions. The presence of a general psychopathology dimension provides a compelling explanation for the observed absence of specificity in etiological mechanisms, biomarkers, and treatment responses across diverse psychiatric diagnoses, underscoring the potential importance of transdiagnostic approaches in advancing psychiatric research and clinical practice (Caspi et al., 2014). A thought-provoking perspective study (Vacca et al., 2025) meticulously examines the changing definitions of “mental disorder” over successive DSM editions, from DSM-III to DSM-5-TR, highlighting persistent conceptual and nosographic inadequacies. The words used in DSM classifications have been very conservative during more than 40 years. They still largely use a descriptive paradigm that focuses on clinically significant suffering, impairment, and inferred malfunction. A major criticism is that basic terms like “dysfunction,” “clinical significance,” and “disturbance” are often used without clear definitions or practical or theoretical boundaries. The authors note that the categorical approach of the DSM tends to make diagnostic categories more rigid, which leads to spurious differences between disorders and higher rates of comorbidity. Moreover, focusing on symptom clusters rather than studying the real causes behind could prevent a deep etiological understanding limiting diagnostic validity. The DSM framework has also some limitations in distinguishing between individual physiological reactions to life events, considered normal, social and cultural situations and abnormal-pathological phenomena. The current risk is to induce people to believe that making normal discomfort seem like a disease or a clinical problem. Even if DSM-5 and DSM-5-TR had more focused on psychological variables and developmental processes, this critical issue has not yet fully addressed. The DSM's nosographic-descriptive paradigm, useful for communication and reliability among different health care professionals, is not enough for detect the complexity, continuity, and contextual embeddedness of psychopathology. It is necessary to promote integrative, holistic, and lifespan-oriented frameworks that go beyond simple descriptive classification, achieving more conceptual coherence and clinical etiology. Recent advancements in psychiatric genomics have increasingly eroded traditional diagnostic boundaries by revealing substantial genetic similarity among mental disorders. Caspi et al. also provide a reliable comprehensive mapping of the shared genetic architecture among major psychopathological problems, utilizing substantial genome-wide association data through the multivariate genomic modeling that identified five higher-order genetic factors able to collectively explain approximately 66% of the heritable variance in psychiatric disorders. These factors classify conditions based on common genetic predispositions rather than symptomatology, consolidating schizophrenia and bipolar disorder, depressive and anxiety disorders including post-traumatic stress disorder, neurodevelopmental disorders such as ADHD and autism, compulsive disorders including obsessive-compulsive disorder and anorexia nervosa, and substance use disorders such as alcohol and nicotine dependence. This framework, based on the latest investigations about genetics, could provide an useful paradigm in order to understand comorbidity, heterogeneity, and overlap among mental diseases based on a neurobiological and physiological basis. Genetic risk factors could be considered in a possible future redefinition of mental disorders (Grotzinger et al., 2025).

## From individual-level approach to a more sustainable population mental health framework about clinical prevention and interventions

There has been a recent emphasis on the need for a revolutionary agenda in psychological science and practice, urging a shift from a focus on individual clinical interventions to a population mental health viewpoint (Dodge et al., 2024). The authors noted that traditional models and settings in psychotherapy, effective at the individual level, could not be the best approach in producing substantial impact on a population dimension, particularly taking into account socioeconomic disparities, climate change, migration, pandemics, and increasing youth morbidity. So a key challenge for current and future clinical psychology is the goal to reformulate the idea of success and outcome, shifting from a “basic symptom relief” logic in treating individuals to measurable improvements in population mental health, taking into account also the reduction in inequities. The authors outline three interconnected strategies: the expansion of evidence-based psychological interventions, the development of community- and policy-level initiatives, and the reform of care systems to create a universal model of primary mental health care. A huge change in the role played by psychotherapy and clinical psychology is requested: it is very important to move from the idea that people, in a sick condition, will meet an health care professional to the best practice of being part of a wider system for preventing illness and improving public health. One of the most relevant problem will be to match and combine traditional health care service with providers who aren't typical in these settings, taking into account the potentiality of digital and low-intensity interventions without reducing quality and clinical impact. The authors noted that it is necessary for mental health services to be included in schools, primary care, and community projects. Innovative infrastructures, financial approaches, and ethical safeguards have to be considered in this innovative proposal in order to achieve universal screening, early prevention, and tiered care models. Only if psychotherapy opens to policy engagement, economic evaluation, and public accountability, areas not been very important for clinical psychology in the past, a paradigm shift will be possible achieving a functional and cost-effective integration between individual therapeutic actions and population-level strategies on a broad scale (Dodge et al., 2024).

## Toward tailored and personalized interventions also very brief if necessary: the case of growing elderly population

A recent policy and practice study (Bannon et al., 2025) particularly focused on the situation of older individuals and their caregivers that are increasing their needs in mental health, taking into account that traditional psychotherapy approaches are not ready for this emerging request. As the world's population gets older, elderly people and informal caregivers deal with a lot of emotional pain, a lot of chronic illnesses, a lot of deteriorating function, and a lot of psychosocial burden. Standard psychotherapy is not always accessible and scalable and has considerable dropout rates in elderly population. The need to modify current care delivery protocols, to reduce long waiting lists and to escalate service demand is urgent. Particularly the authors suggest a single session interventions (SSIs) approach as a viable, low-intensity, and scalable paradigm that can maximize significant clinical impact also in a single encounter. SSIs can be used as stand-alone supports, access points in stepped-care systems, or short-term interventions for people who are waiting for services. Moreover, it is necessary to check accessibility, digital literacy, and quality control overall in case of digital distribution and non-specialist providers. The future of clinical psychology and psychotherapy depends on their capacity to evolve from exclusively high-intensity, specialist-driven models to flexible, population-centered, and system-integrated interventions that prioritize accessibility, efficiency, and immediate impact, while maintaining ethical and scientific integrity (Bannon et al., 2025).

## Some potential risks in our clinical psychology field

“*Too much fragmentation of concepts in psychology, and in clinical psychology too*.”

Psychological research is extremely fragmented, marked by the presence of several concepts and metrics that are never reused and often lack sufficient validation. This fragmentation makes it harder to gather information since it creates redundancies, inconsistencies, and makes it harder to compare studies. Anvari and his co-authors say that the absence of standardization, few barriers to the introduction of new constructs, and incentives that encourage novelty have all led to the uncontrolled spread of *ad hoc* measures, which has caused both jingle and jangle fallacies. To address this problem, the authors propose a coordinated effort to unify psychology and present four interrelated strategies. First, they underline how important it is to stop future fragmentation by utilizing standardized ways to report and assess things, such as the SOBER standards. Second, they agree that using organizational frameworks and semantic modeling tools to show how real and conceptual structures are related is a good idea. Third, the authors underline how crucial it is to constantly improve ideas and measurements based on real-world data. Finally, they propose that committees or communities establish criteria for recognizing and measuring significant psychological concepts. The purpose of all of these ideas is to get the field to stop making too many constructs and start making fewer, more useful, and better validated metrics. This will help psychologists come up with cohesive theories and move further in their field (Anvari et al., 2025).

“*Taking the biopsychosocial model so much for granted that it is neither actively applied nor empirically examined in terms of its actual implementation*.”

Mamtani (2024) notes that the biopsychosocial model (BPSM) has not achieved a general and recognized standardization in ordinary medical and clinical practice in different areas, probably due to the persistent prevalence of the pure biological paradigm. Starting from Engel's fundamental intuition (Engel, 1977), the author underlines that we cannot fully understand and address illness by limiting our focus only on the biological part of the disease. It is relevant for a successful care to take into account patients' psychological experiences and sociocultural contexts. Adding further empirical evidence in BPSM applications can add more credibility in different medical conditions, such as oncological, cardiovascular, pulmonary, gastrointestinal, neurological, and musculoskeletal diseases. Psychosocial (but also cultural and spiritual) aspects have a relevant impact on treatment adherence, quality of life and successful long-term outlook. Other possible reasons of the lack of BPSM use in routine clinical care are worries about the issue that there are no clear clinical criteria and specific guidelines about BPSM. One of the goal of the current and future research is to detect “specific” biopsychosocial models in order to facilitate clinical application in each medical area. Mamtani suggests to improve standardized BPSM-based management procedures, considering also specific training. Moreover, the functional incorporation of digital health technologies could improve cost-effective and scalable biopsychosocial approaches in current clinical care.

Also Bolton (Bolton, 2023) critically re-evaluates the BPSM noting that it has been criticized for not having strong empirical and theoretical foundations. The article discusses the issue that a critical attitude toward a biopsychosocial approach is no longer justified and acceptable, due to the latest advancements in biological, psychological, and social investigations. A unified theoretical basis for a revitalized BPSM, avoiding old and not more reliable reductionist-based explanations, is now available. The study delineates research paradigms congruent with this approach, highlighting findings from psychological therapies, social epidemiology, genetics, and neuroscience that cannot be well-explained by a purely biological model. One typical area with strong biopsychosocial evidences is related to models of chronic stress and pain perception, where there is a lot of scientific demonstrations about dysregulation mechanisms at multiple levels that could promote the onset and continuation of illness and could justify the inclusion of psychosocial interventions, with the biological ones, for a successful care (Bolton, 2023).

Moreover Roberts (2023) explains an interesting theoretical viewpoint about BPSM: philosophers of medicine and health researchers have expressed their criticism about the BPSM limited epistemic and scientific validity. Researchers have not to act as if the model has actual general and total scientific and causal power in each medical field, but they have to demonstrate it in every specific context. Demonstrations are always necessary in this borderline field of medicine-clinical psychology. Researchers are in a conceptual bind because they think BPSM-based approaches will help them understand how diseases work, yet the model itself doesn't allow them any ways to make legitimate or probabilistically sound knowledge assertions. In response, many scholars have unwittingly adopted enduring patterns of flawed reasoning, referred to as “wayward BPSM discourse.” This discourse typically presents outcomes that appear to derive from biopsychosocial analysis. However, a more rigorous examination uncovers dependence on circular reasoning, appeals to authority, and conceptual challenges. The essay employs multiple case studies from the BPSM literature to illustrate the emergence of these argumentative tendencies and examines their broader implications. The development of wayward discourse has made medical research less reliable and could lead to people getting medical care when they don't need it. In order to solve these challenges, we need to put more emphasis on clear ideas and precise rules for how to utilize and interpret the biopsychosocial model. This author also made it clear that “complex interactions” of factors can be part of illness states, but “complex interactions” of elements alone do not make up a disease. It is essential to integrate supplementary and continuous data and evidence for the scientific and clinical validation of the model across multiple domains (Roberts, 2023).

“*Not considering and managing the overwhelming and pervasive role of technology, such as social media, in generating, maintaining, or worsening some psychopathological scenarios in vulnerable subjects*.”

A recent study (Nguyen et al., 2025) underlines the psychological impact of being continuously involved in viewing short-form reel-style films on social media platforms. Through a deep analysis provided with current digital media consumption models, the authors analyze the impact of rapid, algorithm-driven exposure to brief audiovisual content on different users' cognitive processing and general wellbeing. Reel-based video consumption can produce substantial sensory stimulation, immediate satisfaction, and minimal cognitive demand. These results can explain the dangerous effect of habitual usage patterns that can move to an obsessive state. The authors note that watching many reels can generate a distracted mindset, having cognitive (less sustained attention) and emotional (being more emotionally reactive) consequences. Short movies might help users in dealing with tension, boredom, bad feelings, avoiding the possibility to create a more adult way of emotional regulation and mood control. Unfortunately the neurobiological reward mechanisms can motivate users in following with this dangerous behavior of reels consumption, accepted in the current Western social context, also with prolonging usage sessions. We have to take into account also vulnerability traits and factors that can amplify or reduce this mindset, such as impulsivity, emotional dysregulation, and insufficient self-control. The essay situates reel-based video usage within broader contexts of problematic digital media interaction and underscores the imperative for longitudinal and experimental research to clarify causative mechanisms and enduring impacts on mental health and cognitive functioning (Nguyen et al., 2025). A recent study by Casale et al. (2025), based on a longitudinal methodology, revealed temporal correlations among dissociative episodes, maladaptive daydreaming, physical dissociation, and problematic social media usage (PSMU) in young adults. Considering 216 people, the authors found a positive, even if modest, connection among PSMU and several dissociative characteristics, maladaptive daydreaming, and physiological dissociation. The longitudinal approach in this study allows researchers to note that heightened levels of PSMU at baseline might predict an increasing physiological dissociation and absorption/imaginative engagement over time, taking into account that dissociative experiences is not a reliable predictor for subsequent PSMU (Casale et al., 2025). No significant cross-lagged effects were detected for maladaptive daydreaming, dissociative amnesia, or depersonalization/derealization. So a critical level of social media involvement can potentially produce altered states of attention and a progressive separation from physiological experiences toward a possible dissociative tendency. A maladaptive social media use can move to a state of bodily separation creating new emerging problems for clinical psychology and psychotherapy in the area of technology-related addictive behaviors.

## A final remark: evidence based approach is not enough to legitimize psychological treatments in clinical psychology and psychotherapy

As emphasized in Castelnuovo (2010a, 2017), clinical psychological and psychotherapy research must shift its focus from a general demonstration of the efficacy of psychological interventions to the identification of specific treatments for each recognized psychopathology. The inquiry pertains to Paul's initial question: “which treatment, prescribed by whom, and in which circumstances, is the most effective for this particular individual with this specific problem?” [p. 111 (Paul, 1967)]. Unfortunately, many clinical psychologists and psychotherapists are generally reluctant to assess the impact of their clinical practice (Castelnuovo et al., 2016d). Beutler (2009) asserts that “scientists were intentionally obscuring many important results because of an unwarranted devotion to a limited number of scientific methods. Infact, I came to believe that they may be using methods and defining psychotherapy and research informed practice in ways that hindered clinicians from being optimally effective” [p. 301 (Beutler, 2009)]. To enhance scientific recognition and broaden access to psychological therapies (Castelnuovo et al., 2016d), a cost-effective approach must be adopted, extending beyond the clinical efficacy demonstrated by the Empirically Supported Treatments movement (Campbell et al., 2013; Castelnuovo, 2010a,b; Dezetter et al., 2013; Emmelkamp et al., 2014; Mukuria et al., 2013).

According to Castelnuovo et al. (2016), mental health professionals should: “(1) use Research Supported Psychological Treatments as indicated by the Division12-Clinical Psychology of the American Psychological Association (APA) https://www.div12.org/psychologicaltreatments; (2) ensure clinical efficacy through the use of internationally recognized and validated scales; (3) promote cost-benefit analysis, cost-effectiveness analysis and cost-utility analysis using internationally recognized tools, the standardized treatment impact, cost evaluation of health care utilization and productivity loss (absenteeism and presenteeism)…” [p. 2 (Castelnuovo et al., 2016d)].

In conclusion, the future of our discipline (clinical psychology and psychotherapy) toward 2030 will be characterized by a rich set of old and new trends, including the integration of neuroscientific, genetic and transdiagnostic models, the development of digital, Internet and AI-based interventions, and the shift toward personalized, flexible, and scalable protocols. At the same time, another major challenge will included bridging the gap between experimental efficacy and real-world/ecological effectiveness, taking into account an ethical and responsible use of AI-based technologies and algorithms. Other challenges will be overcoming conceptual fragmentation, in some cases generated only by the use of different languages and words used to actually indicate the same concept behind, and translating the biopsychosocial model into reliable, tested and supported clinical practices. Addressing these directions will be essential to move clinical psychology and psychotherapy toward a more significant, sustainable and scientifically grounded role in the mental health system and in the global health care paradigm.
